# Pathology is Always Around Us: Apophenia in Pathology, a Remarkable Unreported Phenomenon

**DOI:** 10.3390/diseases7040054

**Published:** 2019-09-25

**Authors:** Ahmed S. Sultan, Maryam Jessri

**Affiliations:** 1 Department of Oncology and Diagnostic Sciences, School of Dentistry, University of Maryland, Baltimore, MD 21201, USA; 2 School of Dentistry, Australian Centre for Oral Oncology Research & Education, The University of Western Australia, Nedlands, WA 6009, Australia; maryam.jessri@uwa.edu.au

People often wonder, “How do pathologists identify the patterns that lead to a diagnosis?” This is a fundamental question, the answer to which requires significant insight into the psyche of pathologists. Veteran pathologists have long used everyday objects to describe what they see under the microscope to impart their wisdom to their junior colleagues: fried eggs for plasma cells and starry sky appearance for Burkitt lymphoma are two commonly used examples that come to mind. This translation however, is not a unilateral interaction. After long days of staring down upon the pink and purple world of cells, we often begin to recognize that “pathology” follows us home. Many have, while waiting in an elevator or walking, glanced at the tiles on the floor or glimpsed at the patterns on the walls or ceilings, been confronted with a vague semblance of a pathologic entity (Figure 1 and Figure 2). This is not unique to pathologists; as most clinicians and radiologists have at one stage or another, noticed familiar pathognomonic patterns in nature or inanimate objects.

More often than not, clinicians rely on pathologists to confirm their diagnostic suspicions and therefore, pathologists usually are considered the final and defining piece of the diagnostic puzzle. Many pathology departments are inundated with cases and are understaffed to meet the heavy demands. Additionally, there are increasing teaching and administrative responsibilities, which in a competitive market, shift the coveted work-life balance progressively towards work. In these pressing times, when institutions delegate the task of combating burnout to healthcare providers and rely on their resilience, work-related fatigue has become commonplace. The field of pathology has long acknowledged the volatile, uncertain, complex, and ambiguous environment that plagues its members [1]. Given the critical role of pathologists in reaching a final diagnosis, errors in the laboratory can lead to devastating outcomes, jeopardizing our patients’ safety.

If a pathologist can find familiar patterns in bathroom tiles, how can they prevent perceiving non-existing patterns in actual histopathology slides, or in other words how can they avoid falling victim to apophenia [2]? Is this enthusiasm or work-life integration? Could this be an innocent phenomenon that we find humor in, or the first steps of work-related fatigue causing us to hallucinate? Are these “hallucinations” becoming even more commonplace given the high prevalence of burnout among pathologists [3]? Finally, are pathologists more likely to “find” these patterns if they are fatigued or are pathologists pattern finders by nature and nurture?

Most of us have been subject to the preaching of institutionally sponsored speakers cautioning us that in order to do well, one should first feel well. Feeling well however, is not a decision to be made, rather a general reflection of how we fit in, adapt to, and enjoy our environment. While a paternalistic approach will not solve burnout, we should strive to ensure that institutional policies are geared to reducing work-related fatigue. With this, one should acknowledge that both individual and system-wide factors contribute to burnout. In a field, as sensitive as pathology, where erroneous diagnoses can have grave consequences, research by the psychology community should be undertaken to identify early signs of burnout and fatigue. Furthermore, research is needed to delve further into understanding the psyche of pathologists and whether the aforementioned underreported phenomenon is an early indicator of a more insidious process or simply a harmless whimsical afterthought.

## Figures and Tables

**Figure 1:**
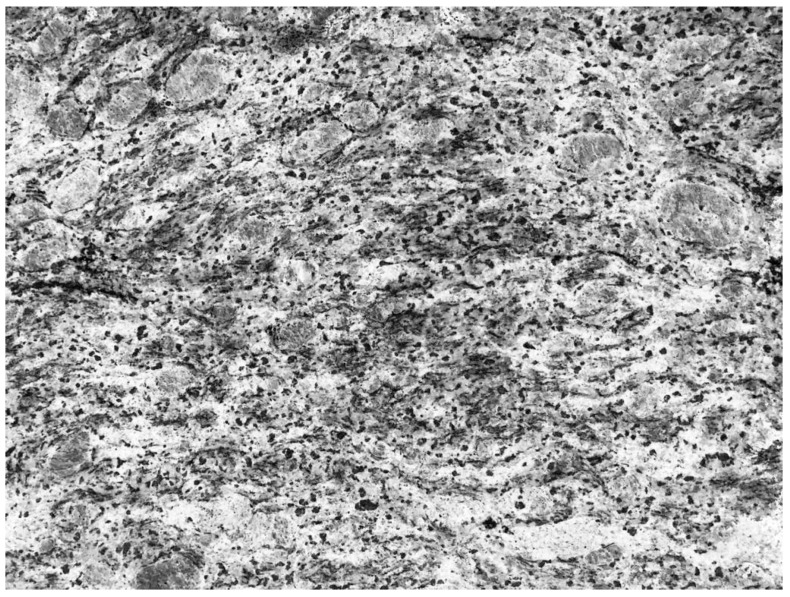
Illustration of a perceived pathological entity of what appears to be a granular cell tumour.

**Figure 2:**
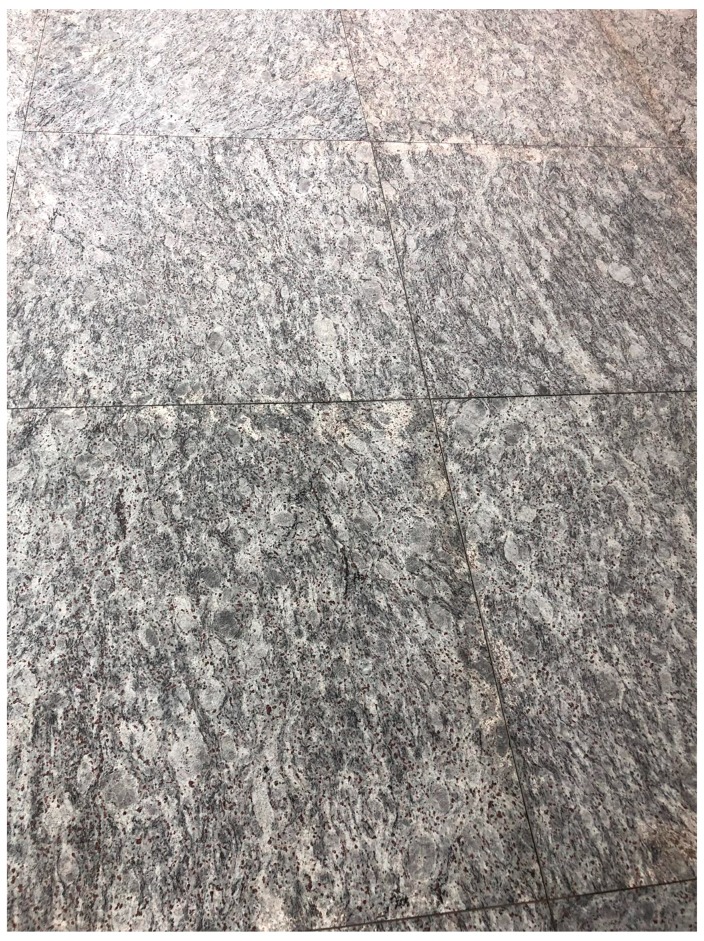
Original zoomed out image of a floor tile that captures one such “hallucination” and features a monochrome image resembling the perceived pathological entity featured in Figure 1.
